# In Vivo Pharmacodynamics of β-Lactams/Nacubactam against Carbapenem-Resistant and/or Carbapenemase-Producing *Enterobacter cloacae* and *Klebsiella pneumoniae* in Murine Pneumonia Model

**DOI:** 10.3390/antibiotics10101179

**Published:** 2021-09-28

**Authors:** Mao Hagihara, Hideo Kato, Toshie Sugano, Hayato Okade, Nobuo Sato, Yuichi Shibata, Daisuke Sakanashi, Jun Hirai, Nobuhiro Asai, Hiroyuki Suematsu, Yuka Yamagishi, Hiroshige Mikamo

**Affiliations:** 1 Department of Molecular Epidemiology and Biomedical Sciences, Aichi Medical University, Nagakute 480-1195, Japan; hagimao@aichi-med-u.ac.jp; 2 Department of Clinical Infectious Diseases, Aichi Medical University, Nagakute 480-1195, Japan; katou.hideo.233@mail.aichi-med-u.ac.jp (H.K.); shibata.yuuichi.414@mail.aichi-med-u.ac.jp (Y.S.); saka74d@aichi-med-u.ac.jp (D.S.); hiraichimed@gmail.com (J.H.); nobuhiro0204@gmail.com (N.A.); hsuemat@aichi-med-u.ac.jp (H.S.); y.yamagishi@mac.com (Y.Y.); 3 Meiji Seika Pharma Co., Ltd., Tokyo 104-8002, Japan; toshie.sugano@meiji.com (T.S.); hayato.okade@meiji.com (H.O.); nobuo.satou@meiji.com (N.S.)

**Keywords:** aztreonam, cefepime, meropenem, nacubactam, carbapenemase-producing Enterobacterales, carbapenem-resistant Enterobacterales, *Enterobacter cloacae*, *Klebsiella pneumoniae*, pneumonia

## Abstract

Carbapenem-resistant Enterobacterales (CRE) and carbapenemase-producing Enterobacterales (CPE) have become global threats. CRE− and CPE− derived infections have been associated with high mortality due to limited treatment options. Nacubactam is a β-lactamase inhibitor and belongs to the new class of diazabicyclooctane. The agent has an in vitro antimicrobial activity against several classes of β-lactamase-producing Enterobacterales. This study evaluated antimicrobial activity of combination therapies including β-lactams (aztreonam, cefepime, and meropenem) and nacubactam against four *Enterobacter cloacae* and six *Klebsiella pneumoniae* isolates with murine pneumonia model. Based on changes in bacterial quantity, antimicrobial activities of some regimens were assessed. Combination therapies including β-lactams (aztreonam, cefepime, and meropenem) with nacubactam showed enhanced antimicrobial activity against CRE *E. cloacae* (−3.70 to −2.08 Δlog_10_ CFU/lungs) and *K. pneumoniae* (−4.24 to 1.47 Δlog_10_ CFU/lungs) with IMP-1, IMP-6, or KPC genes, compared with aztreonam, cefepime, meropenem, and nacubactam monotherapies. Most combination therapies showed bacteriostatic (−3.0 to 0 Δlog_10_ CFU/lungs) to bactericidal (<−3.0 Δlog_10_ CFU/lungs) activities against CRE isolates. This study revealed that combination regimens with β-lactams (aztreonam, cefepime, and meropenem) and nacubactam are preferable candidates to treat pneumonia due to CRE and CPE.

## 1. Introduction

Carbapenem-resistant Enterobacterales (CRE)-caused infections have become a global issue. As therapeutic options are limited, the infections showed high mortality [1,2]. Carbapenemase-producing Enterobacterales (CPE) have spread globally at an alarming rate [3,4]. These bacterial strains have potentials to be highly resistant to not only for carbapenems, but also other commonly used antimicrobials, through the propagation of plasmids encoding carbapenem-hydrolyzing enzymes [5,6].

*Escherichia coli* and *Klebsiella pneumoniae* occupy the majority among detected CPE strains from infected patients [7,8,9]. However, *Enterobacter cloacae* was the second-most detected pathogen with carbapenemase genes in sputum specimens, followed by *K. pneumoniae* [10]. Among Enterobacterales strains, the production of β-lactamases was the main reason for resistance to β-lactams. Then, owing to the hydrolysis of almost all β-lactams, infections due to carbapenemase-producing Enterobacterales strains are the one of the most serious issues to manage in treatment.

Nacubactam (NAC: OP0595) is a novel β-lactamase inhibitor that belongs to non-β-lactam diazabicyclooctane [11]. The agent can inhibit bacterial growth of Enterobacterales expressing some classes of β-lactamases (classes A, C, and D). Comparing protective mechanisms, NAC has similar effects to other β-lactamase inhibitors [11,12,13]. However, the agent has an inhibitory effect of penicillin-binding protein 2 (PBP2) of Enterobacterales too [11]. Therefore, NAC not only has direct antimicrobial effects, but also enhancer effects of co-administered β-lactams against Enterobacterales have several classes of β-lactamases producing genes in an in vitro study [11,12,13].

**Figure 1:**
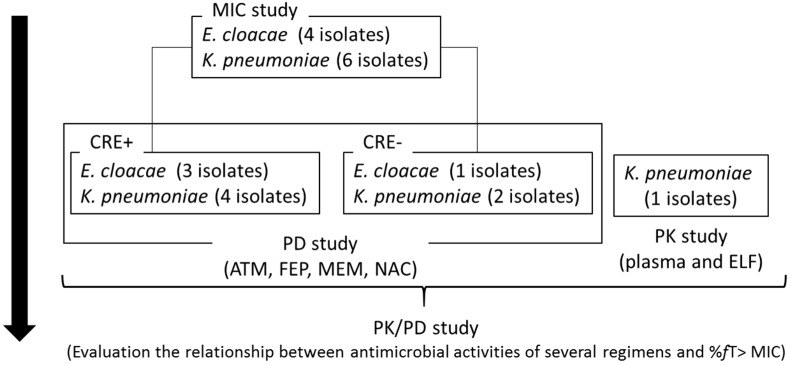
Study flow. MIC: minimum inhibitory concentration; CRE: carbapenem-resistant Enterobacterales; ATM: aztreonam; FEP: cefepime; MEM: meropenem, NAC: nacubactam, PK: pharmacokinetics, PD: pharmacodynamics, ELF: epithelial lining fluid, %*f*T > MIC: the percentage of free drug time above MIC.

In our previous in vivo study with murine thigh infection model, combination therapies including β-lactams (aztreonam (ATM), cefepime (FEP), and meropenem (MEM)) and NAC showed enhanced antibacterial activities against CRE pathogens [14]. However, these combination therapies have not been evaluated the antimicrobial activities against CRE− and/or CPE− derived pneumonia. Therefore, the antimicrobial efficacy of these combination therapies against *E. cloacae* and *K. pneumoniae* were assessed with a murine pneumonia model (Figure 1).

## 2. Results

### 2.1. The Minimum Inhibitory Concentrations (MICs) of ATM, FEP, MEM, and NAC

Table 1 shows MICs of ATM, FEP, MEM, and NAC against the 10 study isolates. Among them, seven isolates are resistance to MEM according to the Clinical and Laboratory Standards Institute (CLSI) breakpoints [15]. Combination regimens of ATM, FEP, and MEM with NAC showed 2- to >128-fold lower MICs, compared with those of β-lactam monotherapies against the CRE+ isolates. Additionally, these combination regimens showed the same to >128-fold lower MICs than those of β-lactam monotherapies against the CRE−/CPE+ isolates. Same MICs were observed between combinations and monotherapies against CRE−/CPE− *E. cloacae* (20-5694).

### 2.2. ATM, FEP, MEM, and NAC Pharmacokinetics (PK) in the Epithelial Lining Fluid (ELF) and Plasma

ATM, FEP, MEM, and NAC PK profiles in ELF and plasma are depicted in Figure S1. In this study, single doses of NAC and each β-lactam monotherapies were used at 100 mg/kg (subcutaneous doses). Table 2 shows the calculated PK parameters of the antimicrobials in plasma and ELF.

### 2.3. Pharmacodynamic (PD) Study with Murine Pneumonia Model

#### 2.3.1. Antimicrobial Efficacies of NAC Monotherapy

At the start of antimicrobial therapies (0 h), bacterial counts in lungs of control mice were between 6.79 to 7.00 log_10_ colony forming units (cfu)/lungs for *E. cloacae* and 6.59 to 7.21 log_10_ cfu/lungs for *K. pneumoniae*. Bacterial number differences of growth control were between 0.06 to 2.58 Δlog_10_ CFU/lungs for *E. cloacae* and −0.77 to 2.74 Δlog_10_ CFU/lungs for *K. pneumoniae* after 24 h from the start of antimicrobial therapies. Bacterial number differences of NAC monotherapy (320, 160, and 80 mg/kg q8h) were between −2.27 and 1.19 Δlog_10_ CFU/lungs for four *E. cloacae* isolates and −1.34 to 2.66 Δlog_10_ CFU/lungs for six *K. pneumoniae* isolates (Figure S2). Among total 10 study isolates, six isolates showed higher bacterial numbers in lungs at 24 h than at 0 h, while maximum dosage of NAC was adopted (320 mg/kg q8h).

#### 2.3.2. Antimicrobial Efficacies against CRE+ Isolates

**Figure 2:**
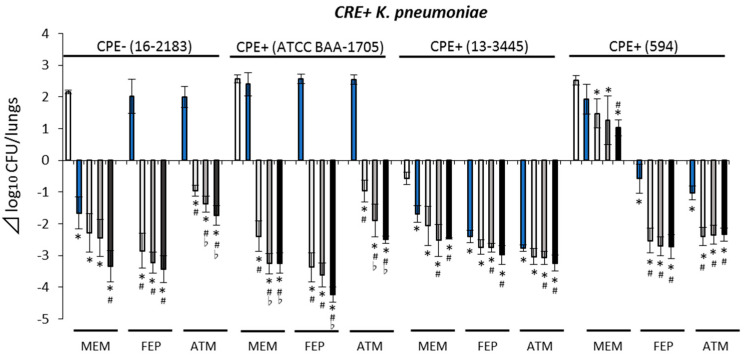
Antimicrobial efficacy of study regimens against CRE+ *Klebsiella pneumoniae* isolates. Control (□), β-lactam (aztreonam, cefepime, meropenem) monotherapy (■), add nacubactam 80 mg/kg q8h (■), add nacubactam 160 mg/kg q8h (■), add nacubactam 320 mg/kg q8h (■). White bar control applies to all the drug combinations shown. *: vs. control *p* < 0.05, ^#^: vs. β-lactam monotherapy *p* < 0.05, ♭: vs. add nacubactam 80 mg/kg q8h *p* < 0.05. CRE: carbapenem-resistant Enterobacterales, CPE: carbapenemase-producing Enterobacterales, Aztreonam: ATM, cefepime: FEP, meropenem: MEM. All data are shown as average ± SD.

**Figure 3:**
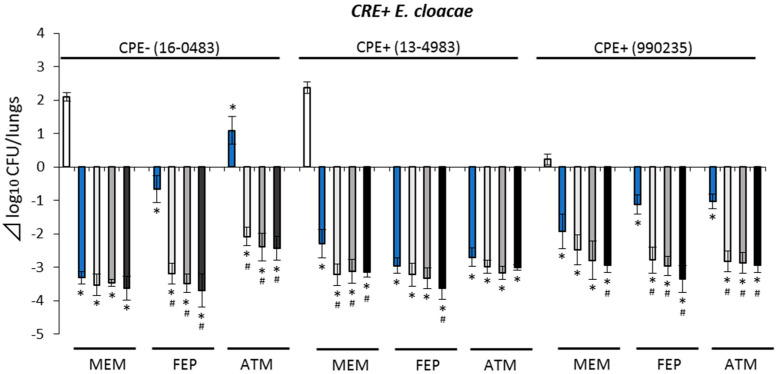
Antimicrobial efficacy of study regimens against CRE+ *Enterobacter cloacae* isolates. Control (□), β-lactam (aztreonam, cefepime, meropenem) monotherapy (■), add nacubactam 80 mg/kg q8h (■), add nacubactam 160 mg/kg q8h (■), add nacubactam 320 mg/kg q8h (■). White bar control applies to all the drug combinations shown. *: vs. control *p* < 0.05, ^#^: vs. β-lactam monotherapy *p* < 0.05. CRE: carbapenem-resistant Enterobacterales, CPE: carbapenemase-producing Enterobacterales, Aztreonam: ATM, cefepime: FEP, meropenem: MEM. All data are shown as average ± SD.

Bacterial number differences in *K. pneumoniae*-infected mice received combination therapies were between −4.24 and 1.47 Δlog_10_ CFU/lungs (Figure 2) and −3.70 and −2.08 Δlog_10_ CFU/lungs for *E. cloacae*-infected mice (Figure 3). Compared with the corresponding β-lactam monotherapies, combination therapies showed higher antimicrobial activities against CRE+ isolates (*p* < 0.05), except when combined with MEM + NAC against CRE+/CPE− isolate (*E. cloacae* 16-0483) and combined with ATM + NAC against CRE+/CPE+ isolate (13-4983). Additionally, the NAC dose-dependent antimicrobial activity of combination therapy with NAC and ATM against CRE+/CPE− isolate (*K. pneumoniae* 16-2183) was observed. When ATM, FEP, and MEM were combined with NAC, similar trends of antimicrobial activity were also observed against CRE+/CPE+ isolate (*K. pneumoniae* ATCC BAA-1705). Moreover, while most combination therapies showed bacteriostatic (−3.0 to 0 Δlog_10_ CFU/lungs) to bactericidal (<−3.0 Δlog_10_ CFU/lungs) activities against CRE isolates, bacterial counts in lungs of MEM + NAC combination therapy group against *K. pneumoniae* (594) were higher at 24 h than at 0 h in the control (>0 Δlog_10_ CFU/lungs).

#### 2.3.3. Antimicrobial Efficacies against CRE− Isolates

**Figure 4:**
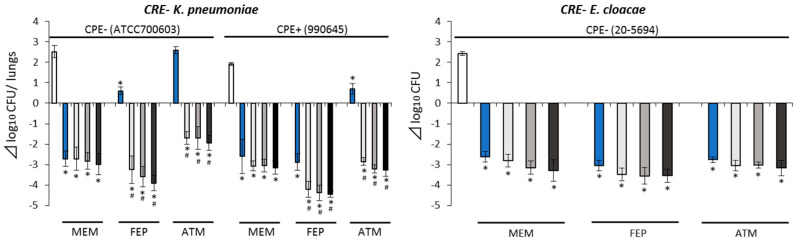
Antimicrobial efficacy of study regimens against CRE− isolates. Control (□), β-lactam (aztreonam, cefepime, meropenem) monotherapy (■), add nacubactam 80 mg/kg q8h (■), add nacubactam 160 mg/kg q8h (■), add nacubactam 320 mg/kg q8h (■). White bar control applies to all the drug combinations shown. *: vs. control *p* < 0.05, ^#^: vs. β-lactam monotherapy *p* < 0.05. CRE: carbapenem-resistant Enterobacterales, CPE: carbapenemase-producing Enterobacterales, Aztreonam: ATM, cefepime: FEP, meropenem: MEM. All data shown as average ± SD.

Bacterial number differences in combination therapies were between −3.56 and −2.81 Δlog_10_ CFU/lungs for *E. cloacae* and −4.46 and −1.70 Δlog_10_ CFU/lungs for *K. pneumoniae* (Figure 4). Comparing with the corresponding β-lactam monotherapies, combination therapies showed enhanced antimicrobial activities, when combined with FEP and AZT with NAC against CRE−/CPE− (*K. pneumoniae* ATCC700603) and CRE−/CPE+ (*K. pneumoniae* 990645). However, NAC did not affect antimicrobial activity of MEM therapy against these isolates. Moreover, no enhanced antimicrobial activity was found in combination therapies against *E. cloacae* (20-5694).

### 2.4. Relationships between Antimicrobial Activities and the Percentage of Free Drug Time above MIC (%fT > MIC)

**Figure 5:**
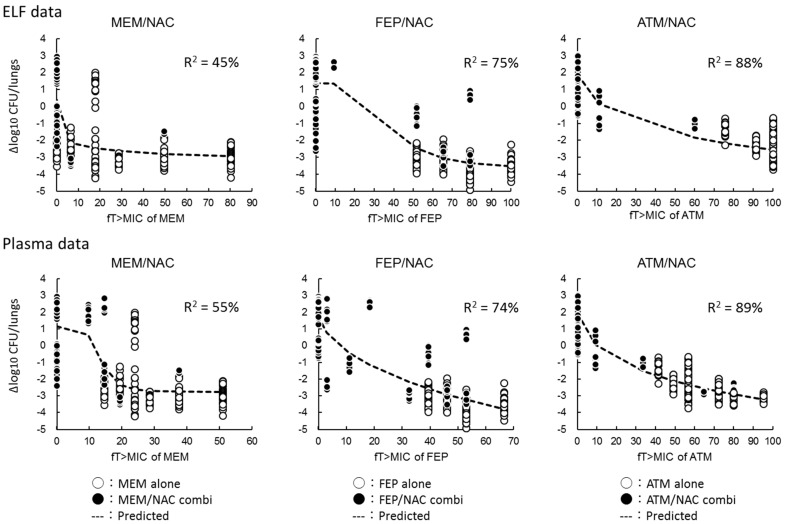
Relationships between %fT > MIC of β-lactams and Δlog_10_ CFU/lungs at 24 h. An inhibitory effect sigmoid Imax model was adapted to reveal the correlations between %fT > MIC of each drug and Δlog_10_ CFU/lungs of each antimicrobial regimen with Phoenix WinNonlin software (ver. 8.1; Certara, L.P.). The R^2^ represents the coefficient of determination. %fT > MIC: the percentage of free drug time above MIC, ATM: aztreonam, FEP: cefepime, MEM: meropenem, NAC: nacubactam.

Figure 5 shows the relationships between Δlog_10_ CFU/lungs and %fT > MIC against study isolates. The %fT > MIC in ELF showed higher correlations with Δlog_10_ CFU/lungs (R^2^ = 0.88 for ATM, 0.75 for FEP, 0.45 for MEM). Similarly, the %fT > MIC in plasma was also correlated with Δlog_10_ CFU/lungs well (R^2^ = 0.89 for ATM, 0.74 for FEP, 0.55 for MEM). Along with the elevation of %fT > MIC value, the bacterial density was reduced after 24 h of antimicrobial treatments.

## 3. Discussion

NAC has a dual mechanism of action. The agent inhibits some classes of serine β-lactamases and PBP2 in Enterobacterales [11,16,17]. In our previous study, the same combination therapies including NAC and β-lactams showed enhanced antimicrobial activities against Enterobacterales have some carbapenemase-producing genes with a murine thigh infection model [14]. However, these combination therapies have not been evaluated against CRE− and CPE−caused pneumonia. In this in vitro study, the combination regimens of β-lactams with NAC showed 1- to >128-fold lower MICs against CPE and CRE isolates, comparing with the corresponding β-lactam monotherapies (Table 1). As previous in vitro study suggested, our results also suggested that NAC enhanced in vitro antimicrobial activities of β-lactams against several classes of β-lactamase-producing *E. cloacae* and *K. pneumoniae* isolates [11,12,13].

Moreover, in this pharmacodynamics evaluation, NAC monotherapy could not show a bactericidal effect against all study isolates (Figure S2). In contrast, combination therapies showed enhanced antimicrobial activities against CRE+ *E. cloacae* and *K. pneumoniae* isolates (Figure 2 and Figure 3). At least one regimen among combination therapies of ATM, FEP, and MEM with NAC showed bactericidal effects against all CRE+ *E. cloacae* and *K. pneumoniae* isolates, excluding the *K. pneumoniae* isolate (594).

Similar to our previous study using a murine thigh infection model [14], combination therapy of MEM and NAC was not effective (>0Δlog_10_ CFU/lungs) against CRE+ *K. pneumoniae* (594) (Figure 2). The isolate has the IMP-6 gene and showed low susceptibility to MEM and NAC, whereas MEM + NAC resulted in relatively lower MIC (2 μg/mL) (Table 1). In vitro studies of the other group conducted showed that the efficacy of NAC is restricted to bacteria with IMP-type carbapenemase-producing genes [11,12,13]. NAC only binds to PBP2. On the other hand, ATM and FEP bind to PBP3 rather than PBP2 [11]. Thus, the NAC-derived enhancer effect was expected when the agent was co-administrated with ATM and FEP than with MEM. Furthermore, we could speculate that NAC was not effective to work as an PBP2 inhibitor to CRE+ *K. pneumoniae* (594) as the isolate has low susceptibility to both MEM and NAC. Although further study to clarify the mechanism is needed, MEM + NAC combination therapy against IMP-type carbapenemase-producing Enterobacterales could be unsuitable, especially when causative bacteria have high MICs (low susceptibilities) of MEM and NAC.

In general, optimizing the antimicrobial dosage according to the PK/PD theory is one of the important components to improve infected patient outcomes. As the PK/PD breakpoint of NAC has not been fully evaluated, it was difficult to interpret our PD study results. However, the antimicrobial activities of β-lactams correlate with %*f*T > MIC well [18,19]. NAC co-administration had no impact on PK profiles of β-lactams (ATM, FEP, and MEM) in plasma [14]. Additionally, we did not observe significant differences in the PK data of plasma between the thigh infection model and pneumonia model [14, Figure S1]. Due to the fact that the reappearance of human PK profiles of antimicrobials was difficult for us, as mice have higher clearance ability than that of humans, we used the same dosage regimens as previous in vivo studies [14] to obtain similar areas under the free drug concentration-time curves (*f*AUC) when β-lactams (ATM, FEP, and MEM) were dosed at 1 g q8h, and NAC (2, 1, and 0.5 g q8h) [20,21,22,23,24,25,26]. Using calculated PK parameters, we conducted PK/PD analysis and found the good relationships between %*f*T > MIC of β-lactams (ATM, FEP, and MEM) in ELF and plasma and antimicrobial activities (Figure 5). Our data will give important insights to explore the optimal dosage regimen for pneumonia patients.

The combination therapy of β-lactams (ATM, FEP, and MEM) and NAC was highly effective against CPE and CRE caused pneumonia. However, we evaluated limited classes of carbapenemases producing Enterobacteriales in this study. In Japan, IMP-1 and -6 type CPE isolates are the most frequently detected [7,8]. However, there are regional differences of the β-lactamases prevalence in Enterobacterales [7,9,27]. Therefore, further studies are needed. As the other limitation, we did not evaluate inoculum effects, while we evaluated antimicrobial activities with similar inoculum size to previous report [28,29,30].

Although our study has a few limitations, this is the first in vivo study to evaluate antimicrobial activities of combination therapies including β-lactams and NAC with pneumonia model. Our PD data suggested combination therapied including NAC is a potent candidate for CRE caused pneumonia. This fact can give new therapeutic choice to antimicrobial treatment for CRE caused pneumonia, while the prevalence and burden of CRE infection are rising. Additionally, we revealed the relationship between these antimicrobial activities and antimicrobial PK data (in plasma and ELF). Therefore, our data would be a preferable reference to explore optimal antimicrobial dosages in the future.

## 4. Materials and Methods

### 4.1. Antimicrobials

To determine the antimicrobial concentrations in ELF and plasma, we used analytical-grade ATM (lot LFSZK: Tokyo Chemical Industry Co., Ltd., Tokyo, Japan), FEP (lot I0L200USP: USP) and MEM hydrate (lot YCY8L: Tokyo Chemical Industry Co., Ltd.). Meiji Seika Pharma Co., Ltd. (Tokyo, Japan) provided us analytical-grade NAC (lot 510-015-4097-01). For all in vivo study, ATM (Eisai Co., Ltd., Tokyo, Japan), FEP (Bristol-Myers Squibb K.K., Tokyo, Japan), MEM (Meiji Seika Pharma Co., Ltd.), and NAC (lot BS1807SB03) were used. Stock solutions of ATM, FEP, and MEM were stored at 4 °C. The stock solutions were reconstituted with normal saline (NS) at desired concentrations.

### 4.2. Microorganisms

Four *E. cloacae* and six *K. pneumoniae* isolates were used in this study (Table 1). Clinical isolates of *E. cloacae* and *K. pneumoniae* were provided from department of Microbial laboratory in Aichi Medical University Hospital. CRE isolates have MEM MIC ≥ 4 μg/mL. CPE isolates have any detectable carbapenemase genes. According to previous our study methods [14], direct sequencing was conducted to screen carbapenemase and other β-lactamase-encoding genes.

### 4.3. Susceptibility Test

The antimicrobial susceptibilities of ATM, FEP, MEM, and NAC were determined with the broth microdilution method, following recommended methods by CLSI [15]. For the combination regimens, the broth microdilution method was conducted in fixed concentration ratios of each β-lactam and NAC (1:1 (*w*/*w*)), according to CLSI method (M100-ED31: Table 5A-2). The β-lactam concentration was used to show MICs in combination therapies. The MIC studies were conducted a minimum of three times, and the geometric MIC was reported.

### 4.4. Animals

Pathogen-free, ICR mice (4-week-old) were acquired from Charles River Laboratories Japan, Inc. (Yokohama, Japan). They were provided food and water ad libitum. They were housed and used following the National Research Council recommendations.

### 4.5. Pneumonia Model

Neutropenic murine pneumonia model was used to evaluate antimicrobial activities of various antimicrobial regimens against *E. cloacae* and *K. pneumoniae* in the lungs of mice [30]. Mice were rendered transiently neutropenic with cyclophosphamide of intraperitoneal doses at Day-4 (150 mg/kg) and Day-1 (100 mg/kg) before inoculation. Study isolates for inoculation were stored at −80 °C. A bacterial suspension was prepared at approximately 10^9^ cfu/mL with study isolates after 24 h incubation of the second transfer. Under anesthesia, mice were orally instilled the bacterial suspension (0.075 mL), and the nares were blocked while being held vertically for 60 s to aspirate the suspension into the lungs. After inoculation, mice were randomly divided into control or each antimicrobial treatment group (*n* = 6, respectively).

### 4.6. PK Studies

For the PK study of ATM, FEP, MEM, and NAC in ELF and plasma, each antimicrobial was dosed subcutaneously (single dose) as monotherapies to the infected mice. The neutropenic pneumonia mice were infected with *K. pneumoniae* ATCC43816. The mice were injected with ATM, FEP, MEM, or NAC (at 100 mg/kg, respectively). Mice were sacrificed at each time point (0.25, 0.5, 1, 2, 3, 4, and 5 h). Then, blood samples from the axillary artery and bronchoalveolar lavage fluid (BALF) samples were collected (*n* = 3 in each group). After the detection of urea concentration in each BALF and plasma sample with a urea assay kit (Urea Assay Kit, BioChain Institute, Inc., Newark, CA, USA), the antimicrobial concentrations in ELF were calculated: ELF antimicrobial concentration = BALF antimicrobial concentration × (the ratio of urea concentrations in plasma/BALF). AUC in ELF and plasma from time 0 to ∞ (AUC0–∞) were calculated according to the trapezoidal method. The PK parameters in the ELF and plasma were calculated with Phoenix WinNonlin software (ver. 8.1; Certara, L.P.).

### 4.7. Instrumentation and Chromatographic Conditions

Liquid chromatography-tandem mass spectrometry (LC-MS/MS) was used to analyze ATM, FEP, MEM, and NAC concentrations at Meiji Seika Pharma Co., Ltd. Detailed conditions and procedures are in the Supplementary document.

### 4.8. PD Study

One hour after inoculation of study isolates, mice received antimicrobials (0.2 mL) subcutaneously (*n* = 6 in each group). We used same dosage of β-lactams and NAC with our previous in vivo study [14] to be similar *f*AUC values of NAC (2.0, 1.0, and 0.5 g), and β-lactams (1.0 g) in blood with clinical PK study data. The following eight-hourly (q8h) doses were evaluated: 700 mg/kg for ATM, 260 mg/kg for FEP, and 140 mg/kg for MEM, 320, 160, and 80 mg/kg for NAC. Control mice were administered NS (0.2 mL).

Twenty-four hours after antimicrobial treatments started, lungs were harvested from mice. Then, mice were sacrificed by CO_2_ exposure. Removed lungs from each mouse were homogenized individually, and the homogenates were plated on trypticase soy agar with 5% sheep blood after serial dilutions with NS to determine the bacterial counts in lungs (cfu/lungs). In addition, six infected mice were harvested just before the start of antimicrobial therapies to know bacterial number in lungs at 0 h. The antimicrobial efficacies were defined as follows: detected bacterial counts in the treated group at 24 h and bacterial counts in the control group at 0 h (Δlog_10_ CFU/lungs).

### 4.9. PK/PD Analysis

The %T > MIC in ELF and plasma for unbound ATM, FEP, MEM, and NAC was calculated with PK parameters (Table S1 and Table S2) in pneumonia mice, serum protein binding ratio [14,20,21,22], and MIC values, with the R software (ver. 3.6.1). For combination therapies, MICs of combinations were used. An inhibitory effect sigmoid *I*_max_ model was adapted to reveal the correlations between %*f*T > MIC of each drug and Δlog_10_ CFU/lungs of each antimicrobial regimen with Phoenix WinNonlin software (ver. 8.1; Certara, L.P.). To estimate the percentage of variance in efficacy, the coefficient of determination (R^2^) was used.

### 4.10. Statistical Analysis

Mann–Whitney U test was used to compare in vitro MIC values of each β-lactam monotherapy and corresponding combination therapies. To compare the in vivo antimicrobial efficacy between the regimens, one-way ANOVA with Bonferroni correction was used. Statistical analysis was performed with JMP, version 10.0 (SAS, Tokyo, Japan). Differences were considered statistically significant at *p* < 0.05.

## 5. Conclusions

Combination therapies including ATM, FEP, and MEM with NAC showed potent in vivo antimicrobial activities against pneumonia caused by CRE and CPE *E. cloacae* and *K. pneumoniae*. These translational data support the potential role of NAC in combination with ATM, FEP, and MEM as a therapy for CRE and CPE *K. pneumoniae* and *E. cloacae* caused human pneumonia.

## Figures and Tables

**Table 1:** Characteristics of *E. cloacae* and *K. pneumoniae* isolates utilized in this study.

Types	Species	Strains	Genotypes	>MIC (μg/mL)						
				MEM	MEM/NAC *	FEP	FEP/NAC *	ATM	ATM/NAC *	NAC
CRE+/CPE+	*E. cloacae*	13-4983 990235	IMP-1, IMP-1	4 64	1 * 8 *	8 64	2 * 4 *	0.12 64	0.03 * 1 *	2 8
	*K. pneumoniae*	ATCC BAA-1705	KPC	8	0.25 *	32	1 *	>128	1 *	2
		13-3445	IMP-1	64	4 *	128	2 *	0.5	0.25 *	2
		594	IMP-6	32	2 *	4	2 *	8	0.25 *	>64
CRE+/CPE−	*E. cloacae*	16-0483		4	2 *	4	1 *	128	2 *	4
	*K. pneumoniae*	16-2183	CTX-M-9	8	2 *	>128	4 *	>128	4 *	>128
CRE−/CPE+	*K. pneumoniae*	990645	DHA-1, IMP-1	0.25	0.25	2	1	64	1	2
CRE−/CPE−	*K. pneumoniae*	ATCC700603	SHV-18	0.03	0.03	1	0.25 *	>128	1 *	4
	*E. cloacae*	20-5694		0.03	0.03	0.03	0.03	0.12	0.12	4

MIC: minimum inhibitory concentration; CPE: carbapenemase-producing Enterobacterales; CRE: carbapenem-resistant Enterobacterales; ATM: aztreonam; FEP: cefepime; MEM: meropenem, NAC: nacubactam. *: *p* < 0.05, comparing MIC values of corresponding β-lactam monotherapy.

**Table 2:** PK parameters in ELF and plasma in murine pneumonia model.

Antimicrobials	Dose (mg/kg)	Tmax (h)	Cmax (µg/mL)	AUC0–∞ (µg h/mL)	T1/2 (h)	Vd/F (L/kg)	CL/F (L/h/kg)
In ELF							
Nacubactam	100	0.5	31	39	1.63	NA	NA
Meropenem	100	0.5	3	6	0.69	NA	NA
Cefepime	100	1	18	32	0.84	NA	NA
Aztreonam	100	0.5	16	27	0.94	NA	NA
In plasma							
Nacubactam	100	0.25	116	131	0.61	0.67	0.76
Meropenem	100	0.25	53	36	0.4	1.6	2.79
Cefepime	100	0.25	80	84	0.41	0.71	1.19
Aztreonam	100	0.5	96	120	0.36	0.43	0.83

PK: pharmacokinetic; ELF: epithelial lining fluid; C_max_: maximum drug concentration; T_max_: time to reach maximum plasma concentration; AUC_0–∞_: AUC in ELF or plasma from time 0 to ∞; T_1/2_: half-life; CL/F: apparent clearance; Vd/F: apparent distribution volume; NA: not applicable.

## Data Availability

The datasets analyzed during this study are available and can be obtained, at request, on reasonable enquiry.

## Supplementary Materials

The following are available online at https://www.mdpi.com/article/10.3390/antibiotics10101179/s1, Figure S1: In vivo pharmacokinetic data of nacubactam, meropenem, cefepime, and aztreonam in ELF and plasma, Figure S2: In vivo efficacy of nacubactam monotherapy, Table S1: Apparent pharmacokinetic parameters in ELF in murine pneumonia model for calculation of *f*T > MIC, Table S2: Pharmacokinetic parameters in plasma in murine pneumonia model for calculation of %*f*T > MIC.

## References

[1] Qureshi Z.A., Paterson D.L., Potoski B.A., Kilayko M.C., Sandovsky G., Sordillo E., Polsky B., Adams-Haduch J.M., Doi Y. (2012). Treatment outcome of bacteremia due to KPC-producing Klebsiella pneumoniae: Superiority of combination antimicrobial regimens. Antimicrob. Agents Chemother..

[2] Tumbarello M., Viale P., Viscoli C., Trecarichi E.M., Tumietto F., Marchese A., Spanu T., Ambretti S., Ginocchio F., Cristini F. (2012). Predictors of mortality in bloodstream infections caused by Klebsiella pneumoniae carbapenemase-producing K. pneumoniae: Importance of combination therapy. Clin. Infect. Dis..

[3] van Duin D., Arias C.A., Komarow L., Chen L., Hanson B.M., Weston G., Cober E., Garner O.B., Jacob J.T., Satlin M.J. (2020). Molecular and clinical epidemiology of carbapenem-resistant Enterobacterales in the USA (CRACKLE-2): A prospective cohort study. Lancet Infect. Dis..

[4] Logan L.K., Weinstein R.A. (2017). The epidemiology of carbapenem-resistant Enterobacteriaceae: The impact and evolution of a global menace. J. Infect. Dis..

[5] Kumarasamy K.K., Toleman M.A., Walsh T.R., Bagaria J., Butt F., Balakrishnan R., Chaudhary U., Doumith M., Giske C.G., Irfan S. (2010). Emergence of a new antibiotic resistance mechanism in India, Pakistan, and the UK: A molecular, biological, and epidemiological study. Lancet Infect. Dis..

[6] Carattoli A., Villa L., Poirel L., Bonnin R.A., Nordmann P. (2012). Evolution of IncA/C blaCMY-_2_-carrying plasmids by acquisition of the blaNDM-_1_ carbapenemase gene. Antimicrob. Agents Chemother..

[7] Yamamoto N., Asada R., Kawahara R., Hagiya H., Akeda Y., Shanmugakani R.K., Yoshida H., Yukawa S., Yamamoto K., Takayama Y. (2017). Prevalence of, and risk factors for, carriage of carbapenem-resistant Enterobacteriaceae among hospitalized patients in Japan. J. Hosp. Infect..

[8] Asai N., Sakanashi D., Suematsu H., Kato H., Hagihara M., Nishiyama N., Koizumi Y., Yamagishi Y., Mikamo H. (2018). The epidemiology and risk factor of carbapenem-resistant enterobacteriaceae colonization and infections: Case control study in a single institute in Japan. J. Infect. Chemother..

[9] Xu Y., Gu B., Huang M., Liu H., Xu T., Xia W., Wang T. (2015). Epidemiology of carbapenem resistant Enterobacteriaceae (CRE) during 2000-2012 in Asia. J. Thorac. Dis..

[10] Cai Z., Tao J., Jia T., Fu H., Zhang X., Zhao M., Du H., Yu H., Shan B., Huang B. (2020). Multicenter Evaluation of the Xpert Carba-R Assay for Detection and Identification of Carbapenemase Genes in Sputum Specimens. J. Clin. Microbiol..

[11] Morinaka A., Tsutsumi Y., Yamada M., Suzuki K., Watanabe T., Abe T., Furuuchi T., Inamura S., Sakamaki Y., Mitsuhashi N. (2015). OP0595, a new diazabicyclooctane: Mode of action as a serine β-lactamase inhibitor, antibiotic and β-lactam ‘enhancer’. J. Antimicrob. Chemother..

[12] Livermore D.M., Warner M., Mushtaq S., Woodford N. (2016). Interactions of OP0595, a novel triple-action diazabicyclooctane, with β-lactams against OP0595-resistant Enterobacteriaceae mutants. Antimicrob. Agents Chemother..

[13] Livermore D.M., Mushtaq S., Warner M., Woodford N. (2015). Activity of OP0595/β425 lactam combinations against Gram-negative bacteria with extended-spectrum, AmpC and carbapenem-hydrolysing β-lactamases. J. Antimicrob. Chemother..

[14] Hagihara M., Kato H., Sugano T., Okade H., Sato N., Shibata Y., Sakanashi D., Asai N., Koizumi Y., Suematsu H. (2021). Pharmacodynamic evaluation of meropenem, cefepime, and aztreonam combined with a novel β-lactamase inhibitor, nacubactam, against carbapenem-resistant and/or carbapenemase-producing Klebsiella pneumoniae and Escherichia coli using a murine thigh infection model. Int. J. Antimicrob. Agents.

[15] Clinical and Laboratory Standards Institute (2018). Performance Standards for Antimicrobial Susceptibility Testing.

[16] Docquier J.D., Mangani S. (2018). An update on β-lactamase inhibitor discovery and development. Drug Resist. Updat..

[17] Mushtaq S., Vickers A., Woodford N., Haldimann A., Livermore D.M. (2019). Activity of nacubactam (RG6080/OP0595) combinations against MBL-producing Enterobacteriaceae. J. Antimicrob. Chemother.

[18] Li C., Du X., Kuti J.L., Nicolau D.P. (2007). Clinical pharmacodynamics of meropenem in patients with lower respiratory tract infections. Antimicrob. Agents Chemother..

[19] Craig W.A., Redington J., Ebert S.C. (1991). Pharmacodynamics of amikacin in vitro and in mouse thigh and lung infections. J. Antimicrob. Chemother.

[20] Sumita Y., Nouda H., Tada E., Kohzuki T., Kato M., Okuda T., Fukasawa M. (1992). Pharmacokinetics of meropenem, a new carbapenem antibiotic, parenterally administered to laboratory animals. Chemotherapy.

[21] Hirano M., Masuyoshi S., Kondo S., Asai Y., Oki T. (1991). Distribution, metabolism and excretion of cefepime in rats. Chemotherapy.

[22] Kita Y., Fugono T., Imada A. (1986). Comparative pharmacokinetics of carumonam and aztreonam in mice, rats, rabbits, dogs, and cynomolgus monkeys. Antimicrob. Agents Chemother..

[23] Nakajima M., Uematsu T., Kanamaru M., Ueno K. (1992). Phase I Study of Meropenem. Chemotherapy.

[24] Nakajima M., Uematsu T., Kanamaru M., Chuna I., Kidono M., Shimizu K. (1991). Phase I Study of Cefepime (BMY-28242): Single Dose Study in Healthy Male Volunteers. Chemotherapy.

[25] Nakashima M., Uematsu T., Takiguchi Y., Maeda Y. (1985). Pharmacokinetics and Safety of Azthreonam in Healthy Japanese Volunteers. Jpn. Pharmacol. Ther..

[26] Mallalieu N.L., Winter E., Fettner S., Patel K., Zwanziger E., Attley G., Rodriguez I., Kano A., Salama S.M., Bentley D. (2020). Safety and Pharmacokinetic Characterization of Nacubactam, a Novel β-Lactamase Inhibitor, Alone and in Combination with Meropenem, in Healthy Volunteers. Antimicrob. Agents Chemother..

[27] Arakawa Y. (2015). Global spread of multidrug-resistant microbes including CRE and clinical alerts. Jpn. J. Chemother..

[28] Lasko M.J., Abdelraouf K., Nicolau D.P. (2021). In Vivo Activity of WCK 4282 (High-Dose Cefepime/Tazobactam) against Serine-β-lactamase-Producing Enterobacterales and Pseudomonas aeruginosa in the Neutropenic Murine Lung Infection Model. Antimicrob. Agents Chemother..

[29] Maglio D., Ong C., Banevicius M.A., Geng Q., Nightingale C.H., Nicolau D.P. (2004). Determination of the in vivo pharmacodynamic profile of cefepime against extended-spectrum-beta-lactamase-producing Escherichia coli at various inocula. Antimicrob. Agents Chemother..

[30] Tessier P.R., Keel R.A., Hagihara M., Crandon J.L., Nicolau D.P. (2012). Comparative in vivo efficacies of epithelial lining fluid exposures of tedizolid, linezolid, and vancomycin for methicillin-resistant Staphylococcus aureus in a mouse pneumonia model. Antimicrob. Agents Chemother..

