# Supplementary material for: In Vivo Pharmacodynamics of β-Lactams/Nacubactam against Carbapenem-Resistant and/or Carbapenemase-Producing Enterobacter cloacae and Klebsiella pneumoniae in Murine Pneumonia Model
Source: Antibiotics (Basel). 2021 Sep 28;10(10):1179. doi: 10.3390/antibiotics10101179 (PMC8532682; doi:10.3390/antibiotics10101179)
Supplement: Supplementary file 1 [file antibiotics-10-01179-s001.zip › antibiotics-1375184-supplementary.pdf]

antibiotics

MDPI

Article

In Vivo Pharmacodynamics of β-Lactams/Nacubactam against Carbapenem-Resistant and/or Carbapenemase-Producing *Enterobacter cloacae* and *Klebsiella pneumoniae* in Murine Pneumonia Model

Mao Hagihara [1,2], Hideo Kato [2], Toshie Sugano [3], Hayato Okade [3], Nobuo Sato [3], Yuichi Shibata [2], Daisuke Sakanashi [2], Jun Hirai [2], Nobuhiro Asai [2], Hiroyuki Suematsu [2], Yuka Yamagishi [2] and Hiroshige Mikamo [2,*]

1 Department of Molecular Epidemiology and Biomedical Sciences, Aichi Medical University, Nagakute 480-1195, Japan; hagimao@aichi-med-u.ac.jp

2 Department of Clinical Infectious Diseases, Aichi Medical University, Nagakute 480-1195, Japan; katou.hideo.233@mail.aichi-med-u.ac.jp (H.K.); shibata.yuuichi.414@mail.aichi-med-u.ac.jp (Y.S.); saka74d@aichi-med-u.ac.jp (D.S.); hiraichimed@gmail.com (J.H.); nobuhiro0204@gmail.com (N.A.); hsuemat@aichi-med-u.ac.jp (H.S.); y.yamagishi@mac.com (Y.Y.)

3 Meiji Seika Pharma Co., Ltd., Tokyo 104-8002, Japan; toshie.sugano@meiji.com (T.S.); hayato.okade@meiji.com (H.O.); nobuo.satou@meiji.com (N.S.)

* Correspondence: mikamo@aichi-med-u.ac.jp; Tel/Fax: +81-561-61-1842

Supplemental document

Sample Preparation, Instrumentation and Chromatographic Conditions to Detect Antimicrobial Concentrations.

For analyzing nacubactam (NAC), meropenem (MEM), cefepim (FEP), and aztreonam (ATM) concentrations, liquid chromatography-tandem mass spectrometry (LC-MS/MS) was used. Formic acid and Ammonium formate (FUJIFILM Wako Pure Chemical Corporation, Osaka, Japan), dibutylammonium acetate (Tokyo Chemical Industry Co., Ltd., Tokyo, Japan), acetonitrile (Nacalai Tesque, Inc., Kyoto, Japan), methanol (Nacalai Tesque, Inc.), 2-propanol (FUJIFILM Wako Pure Chemical Corporation), 3-(*N*-Morpholino)propanesulfonic acid (MOPS) or (3-(*N*-Morpholino)propanesulfonic acid sodium salt(MOPS-Na) (Nacalai Tesque, Inc.) were purchased. Tap water was purified using an ultrapure water production system, Milli-Q Q-POD™ (Nihon Millipore K.K.), to produce Milli-Q water for use.

Standard stock solutions of MEM were prepared at 1.0 mg/mL in 0.1 M MOPS buffer (pH 7.0). NAC and ATM were prepared at 10 mg/mL in 50% acetonitrile. FEP was prepared at 1 mg/mL in distilled water. The IS stock solutions of biapenem (1.0 mg/mL), cephalexin hydrate (1.0 mg/mL) and ceftazidime hydrate (1.0 mg/mL) were prepared in distilled water.

An aliquot (50 or 25 μL) of the plasma or BALF sample was added to acetonitrile, methanol and biapenem, cephalexin or ceftazidime as internal standards (ISs). These samples were vortexed-mixed and centrifuged for 5 min at 10,000 × g and 4 °C. The supernatant was filtrated through a centrifugal filter unit, and a portion of the filtrate was diluted with purified water (× 2). The sample was analyzed by a high-performance liquid chromatography with tandem mass spectrometry (LC-MS/MS). LC-MS/MS analyses were performed on a QTRAP® 5500 and 6500+ System (AB Sciex Pte. Ltd).

For the determination of NAC and ATM, chromatographic separation was performed using a reversed-phase HPLC column (AQUITY UPLC HSS T3, 100 × 2.1 mm i.d., 1.8 μm, Waters, Tokyo, Japan) with 5 mM dibutylannmonium acetate (A) and 0.1% formic acid in acetonitrile (B) as the mobile phase under a gradient condition at a flow rate of 0.3

mL/min. A linear gradient was applied as follows: hold isocratic at 0% B, 0.0–0.5 min; increase from 0 to 70% B, 0.5–3.0 min; hold isocratic at 70% B, 3.0–3.5 min; decrease from 70 to 0% B, 3.5–3.51 min; re-equilibrate at 0% B, 3.51–6.1 min. The temperatures of the column oven and sample compartment were set at 35°C and 10°C, respectively. The mass spectrometer with a Turbo V™ Source was operated in a negative ionization mode. The heater gas temperature was set at 500 °C, and the ionspray voltage was set at –4,500 V. The analytes (NAC and ATM) and the IS (cephalexin) were detected using multiple reaction monitoring (MRM) mode by monitoring the transitions: m/z 323 → m/z 96 for NAC, m/z 434 → m/z 96 for ATM and m/z 346 → m/z 268 for cephalexin. For quantification, the calibration curve was constructed using linear least-squares regression of the peak area ratios of analyte to the IS versus the analyte concentrations with a $1/y^2$ weighting factor. The analyte concentrations in samples were determined by interpolating the peak area ratios of analyte to the IS on the calibration curve. The Lower limit of quantification (LLOQ) was 10 ng/mL for NAC and ATM.

For the determination of FEP, chromatographic separation was performed using a reversed-phase HPLC column (CAPCELL PAK INERT ADME-HR, 50 × 2.0 mm i.d., 3 µm, OSAKA SODA, OSAKA, Japan) with 10 mM ammonium formate in 0.1% formic acid (A) and acetonitrile (B) as the mobile phase under a gradient condition at a flow rate of 0.4 mL/min. A linear gradient was applied as follows: hold isocratic at 1% B, 0.0–0.5 min; increase from 1 to 90% B, 0.5–3.0 min; hold isocratic at 90% B, 3.0–4.0 min; decrease from 90 to 1% B, 4.0–4.1 min; re-equilibrate at 1% B, 4.1–5.5 min. The temperatures of the column oven and sample compartment were set at 40 °C and 10 °C, respectively. The mass spectrometer with a Turbo V™ Source was operated in a positive ionization mode. The heater gas temperature was set at 500 °C, and the ionspray voltage was set at 4,500 V. The analyte (FEP) and the IS (ceftazidime) were detected using MRM mode by monitoring the transitions: m/z 481 → m/z 396 for FEP and m/z 548 → m/z 469 for ceftazidime. Data acquisition and quantification were performed as described above. The LLOQ was 10 ng/mL for FEP.

For the determination of MEM, chromatographic separation was performed using a reversed-phase HPLC column (ACQUITY UPLC BEH C_{18}, 50 × 2.1 mm i.d., 1.7 µm, Waters, Tokyo, Japan) with 0.1% formic acid (A) and acetonitrile (B) as the mobile phase under a gradient condition at a flow rate of 0.4 mL/min. A linear gradient was applied as follows: hold isocratic at 1% B, 0.0–0.5 min; increase from 1 to 50% B, 0.5–3.0 min; increase from 50 to 80% B, 3.0–3.1 min; hold isocratic at 80% B, 3.1–3.9 min; decrease from 80 to 1% B, 3.9–4.0 min; re-equilibrate at 1% B, 4.0–5.0 min. The temperatures of the column oven and sample compartment were set at 25 °C and 4 °C, respectively. The mass spectrometer with a Turbo V™ Source was operated in a positive ionization mode. The heater gas temperature was set at 500 °C, and the ionspray voltage was set at 4,500 V. The analyte (MEM) and the IS (biapenem) were detected using MRM mode by monitoring the transitions: m/z 384 → m/z 141 for MEM and m/z 351 → m/z 265 for biapenem. Data acquisition and quantification were performed as described above. The LLOQ was 2.5 ng/mL for MEM.

Pharmacokinetic Parameters in ELF or Plasma in Murine Pneumonia Model for Calculation of T>MIC

ELF or plasma concentrations of nacubactam, meropenem, cefepime or aztreonam in murine pneumonia after subcutaneous administration were analyzed with 1- or 2-compartment model with constant IV infusion, because none or only one ELF or plasma concentration were determined in the absorption phase. The obtained PK parameters were used for the calculation of *f*T>MIC in ELF and plasma.

Supplemental Tables

Table S1. Apparent pharmacokinetic parameters in ELF in murine pneumonia model for calculation of *f*T>MIC.

Antimicrobials	V (L/kg)	K_{10} (hr^{-1})
Nacubactam	2.76	0.978
Meropenem	21.8	0.846
Cefepime	4.97	0.640
Aztreonam	6.66	0.552

Table S2. Pharmacokinetic parameters in plasma in murine pneumonia model for calculation of *f*T>MIC.

Antimicrobials	V_1 (L/kg)	K_{10} (hr^{-1})	K_{12} (hr^{-1})	K_{21} (hr^{-1})	PBR (%)
Nacubactam	0.672	1.20	0.000018	2.47	0.0 [14]
Meropenem	1.48	1.92	0.000024	0.0193	33.8 [20]
Cefepime	0.986	1.27	0.000039	1.24	29.0 [21]
Aztreonam	0.767	1.13	0.000036	0.162	84.0 [22]

PBR: protein binding ratio.

Supplemental Figures

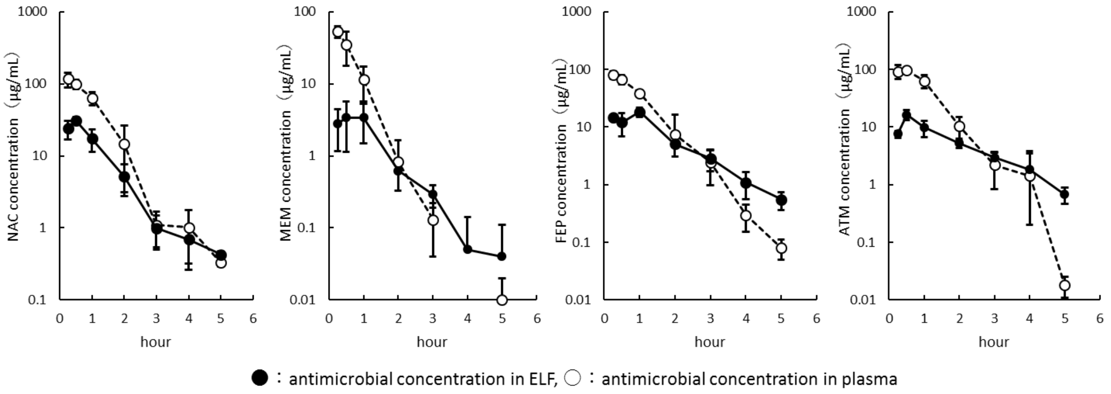

Figure S1. In vivo pharmacokinetic data of nacubactam, meropenem, cefepime, and aztreonam (□) in ELF and plasma. Antimicrobials were administered at a dose of 100 mg/kg. ●: nacubactam (NAC), meropenem (MEM), cefepime (FEM), or aztreonam (ATM) monotherapy pharmacokinetics data in epithelial lining fluid (ELF). ○: nacubactam (NAC), meropenem (MEM), cefepime (FEM), or aztreonam (ATM) monotherapy pharmacokinetics data in plasma.

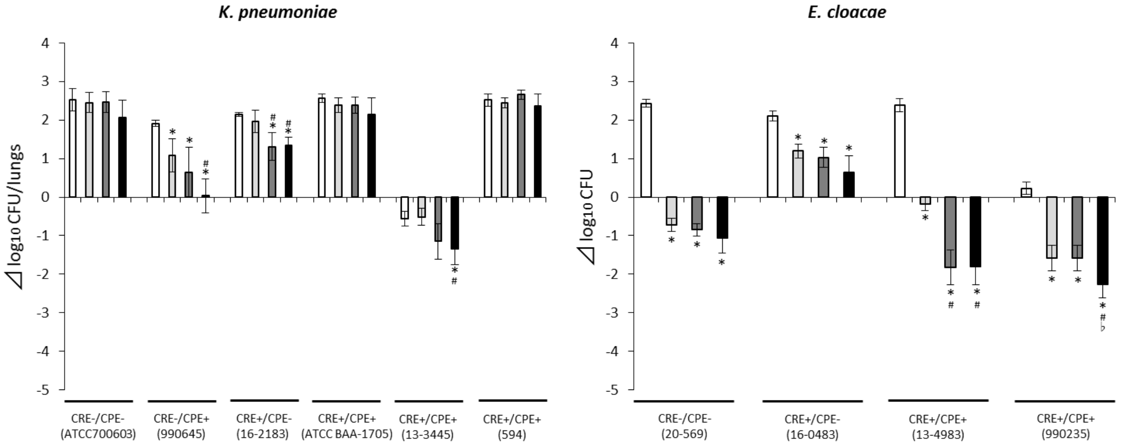

Figure S2. In vivo efficacy of nacubactam monotherapy. □： control, ■: nacubactam 80 mg/kg q8h, ■： nacubactam 160 mg/kg q8h, ■： nacubactam 320 mg/kg q8h. *： vs. control $p < 0.05$, $^{\sharp}$： vs. nacubactam 80 mg/kg q8h $p < 0.05$, $^{\flat}$： vs. nacubactam 160 mg/kg q8h $p < 0.05$.
